# 

**Published:** 1896-05

**Authors:** 


### Notice.

The twenty-eighth annual meeting of the New York State Dental Society will be held in Albany, May 13th and 14th, 1896, in Geological Hall. Quite a full programme of very interesting subjects has been prepared. Papers will be presented upon the following subjects succeeding the annual address of the President, Dr. H. J. Burkhart, of Batavia, N. Y.:

"Pyorrhea Alveolaris, Its Causation, Diagnosis and Treatment," by C. N. Peirce, of New York. Discussion opened by G. S. Allan, of New York.

"Professional Fees," by S. G. Perry, of New York. Discussion opened by Wm. Jarvie, of Brooklyn.

"The Application of Medicaments in Pathological Conditions of the Oral Cavity," by M. W. Foster, Baltimore. Discussion opened by R. H. Hofheinz, Rochester.

"The Latest Achievements in Dental Art," by R. Ottolengui, Correspondent.

Report of Committee on Practice, by M. L. Rhein, Chairman.

The State Board of Dental Examiners will be in session at the same time, of which Dr. Wm. Carr, of New York, is Chairman.

The New York Society is one of our best State societies, and none can be present without being benefited.

### Notice.

The Kentucky State Dental Association will hold its annual meeting at Louisville, June 16th to 19th, 1896.

A cordial invitation is extended to members of the profession in good standing to be present.

The State Board of Examiners will meet at the same time and place for the examination of candidates and such other business as may come before it.

J. H. Baldwin, Secretary.

### Notice.

The third union meeting of the Maryland State Dental Association and the Washington City Dental Society will be held May 8th and 9th, 1896, at Columbian Dental College Building,

Washington, D. C. Standing committees will report upon the following subjects:

"On Publication and Voluntary Essays," by A. W. Sweeny, Chairman, of Washington.

"Some Notes on Gold as a Filling Material," by L. Ashley Faught, of Philadelphia.

"Dermoid Cysts vs. Migrated Teeth—a Criticism," by Dr. Wm. A. Mills, of Baltimore.

"Curretting of Alveolar Cysts," by Dr. D. E. Wiber, of Washington.

"Anatomy, Physiology and Histology," by W. S. Twilley, of Baltimore, Chairman. Reported by H. C. Thompson, of Washington.

"Operative Dentistry," reported by C. M. Gingrich, Chairman, of Baltimore.

Essay, by L. C. F. Hugo, of Washington, "Approximal Work as Determining the Shape of the Inter-dental Space—the Important Factor Involved."

"Prosthetic Dentistry," report by J. Rowland Walton, Chairman, of Washington.

"Dental Education, Literature and Nomenclature," reported F. J. S. Gorgas, Chairman, of Baltimore.

Essay by Wm. A. Mills, of Baltimore, "A Plea for a Higher Education of Individual Members of the Dental Profession."

"Pathology and Therapeutics," reported by L. F. C. Hugo, Chairman, of Washington.

"Pathology and Therapeutics of Dead Teeth," by Gordon H. Claude, of Annapolis, Maryland.

"Treatment of Oral Acidity with Milk Magnesia," by Dr. Wm. S. Donnelly, of Washington.

"Crown and Bridge-Work," reported by W. B. Finney, Chairman, of Baltimore.

"Orthodontia and Dental Appliances," reported by M. F. Finley, of Washington.

"Oral Surgery," reported by P. E. Sasscer, Chairman, of Waldorf, Maryland.

"Report of a Peculiar Case of Replantation," by R. H. Jones, of Wilmington, Delaware.

"Report of a Case of Fracture of the Lower Maxillary Bone," by J. R. Hagan, of Washington.

"Report of Case Restoring Oral Cavity from Ravages of Epithelioma," by David Genese, of Baltimore.

"Report of Two Cases of Epulic Tumor," by M. F. Finley, of Washington.

"Anæsthetics," reported by L. L. Harban, Chairman, of Washington.

"Hygiene," reported by H. W. Lakin, Chairman, Boonesboro, Maryland.

Essay, by J. H. P. Benson, of Washington, "Lectures upon Dental Hygiene in our Public Schools and Colleges."

"Dental Legislation," reported by H. B. Noble, Chairman, of Washington.

Paper by Richard Grady, of Baltimore, "A History of Maryland's New Dental Law."

Essay by A. W. Sweeny, of Washington, "Dental Legislation."

Paper by H. B. Noble, of Washington, "Recent Legislation in the District of Columbia."

Extensive arrangements have been made for interesting clinics and demonstrations which will embrace a large number of subjects.

### EDITORIAL.

### THE DENTAL REGISTER,

### A MONTHLY MAGAZINE DEVOTED TO THE INTERESTS OF THE DENTAL PROFESSION.

*Terms Reduced to $2.00 Per Year. Single Copies, 25 Cents.*

### EDITED BY PROF. J. TAFT, D.D.S.

—AND—

### Published by SAM'L A. CROCKER & CO., Ohio Dental and Surgical Depot.

The volume commences in January and closes in December.

The REGISTER being issued monthly is always fresh, therefore Indispensable to the practitioner who desires to keep posted up with the new inventions and improvements which are daily developed. Neither expense nor effort will be spared to give value and variety to its contents. Articles will be illustrated with well executed engravings whenever they will add to the interest or assist to explanation.

Its extensive circulation and frequency of issue make it one of the BEST DENTAL ADVERTISING MEDIUMS in the United States.

All subscriptions must begin with January or July.

Local or special notices, five lines or less, will be inserted for $3 each insertion; over five lines, 50 cts. per line.

All advertising bills payable in advance, or at furthest, after the issue of the first number containing the advertisement. All transient advertisements to be paid for in advance.

☞CONTRIBUTIONS SOLICITED.

Exclusively Editorial Communications should be addressed to the Editor.

The latest number of the REGISTER may be ordered with or without other goods at any time, and all letters should be addressed to [illegible]

